# Memory Improvement Effect of Ethanol Garlic (*A. sativum*) Extract in Streptozotocin-Nicotinamide Induced Diabetic Wistar Rats Is Mediated through Increasing of Hippocampal Sodium-Potassium ATPase, Glutamine Synthetase, and Calcium ATPase Activities

**DOI:** 10.1155/2017/3720380

**Published:** 2017-12-27

**Authors:** Ibrahim Semuyaba, Abass Alao Safiriyu, Emmanuel Ayikobua Tiyo, Remón Figueredo Niurka

**Affiliations:** Department of Physiology, Faculty of Biomedical Sciences, Kampala International University, Western Campus, Bushenyi, Uganda

## Abstract

Studies suggest that garlic (*A. sativum*) improves memory dependent on the hippocampus. However, the effect of ethanol garlic extract on hippocampus Na^+^/K^+^ ATPase, Ca^2+^ ATPase, and glutamine synthetase (GS) activities as possible mechanisms in memory improvement in diabetic Wistar rats has not been reported. Twenty-four male Wistar rats weighing 200–250 g were divided into three groups with 8 rats each. Group (A), normal control rats, and Group (B), diabetic rats, received 1 ml of normal saline; diabetic rats in Group (C) received 1000 mg/kg of garlic extract orally for 21 days. Hyperglycemia was induced by a single intraperitoneal injection of streptozotocin 60 mg/kg followed by 120 mg/kg nicotinamide while extraction of garlic was done by cold maceration method. Memory was tested in all groups. After that, the rats were sacrificed, the brain was removed, and the hippocampi were carefully excised and then homogenized. Activities of Na^+^/K^+^ ATPase, calcium ATPase, and GS were analyzed from the homogenate. Results showed improvement in memory and a significant increase (*P* < 0.05) in hippocampus Na^+^/K^+^ ATPase, Ca^2+^ ATPase, and GS activities in diabetic rats treated with garlic extract. In conclusion, the increased activity of hippocampus Na^+^/K^+^ ATPase, calcium ATPase, and glutamine synthetase may account for the memory improvement.

## 1. Introduction

Diabetes mellitus is a metabolic disease which leads to serious neuronal damage and cognitive deficits. It is considered as one of the causes of progressive neurodegeneration, with impairment in memory as a major complication [1]. This is shown by impairment in decision making and judgment especially in patients with this condition [2]. Elevated blood glucose levels in diabetic rats have been shown to impair memory by causing enhanced production of free radicals [3]. Studies have reported the alteration in transmembrane fluidity, affecting the activity of membrane receptors and inhibiting Na^+^/K^+^ ATPase, Ca^2+^ ATPase [4], and glutamine synthetase [5].

Membrane bound enzymes Na^+^/K^+^ ATPase and calcium ATPase are important in neuronal excitability and neurotransmitter release. These enzymes maintain concentration gradients of Na^+^, K^+^, and Ca^2+^ across the cell membrane. Inhibition of these enzymes leads to impairment in learning and memory [6]. Abnormalities in intracellular calcium ion regulation leads to neuronal death and impairment in neurological function [7]. In diabetic rat whole brain, suppression of Ca^2+^ ATPase and Na^+^/K^+^ ATPase activities has been reported [8]. Glutamine synthetase is an enzyme important in controlling intracellular concentration of glutamate. It is suggested that accumulation of glutamate in the extracellular fluid causes a decrease in glutamine synthetase which may lead to seizures [9]. It has been reported that when there is inhibition of glutamine synthetase, neurotransmission at the synapse in the retina is affected [10].

Herbs as an alternative treatment of DM especially in poor countries have been utilized more frequently in treating patients with diabetes. World Health Organization indicated that more than 80% of the people in the world trust use of herbal medicines [11, 12] and in developing countries 3.5 billion are estimated to use medicinal plants [13]. Garlic as one of the herbs has been shown to have numerous medicinal properties. It has been suggested to have antiangiogenic effects [14] and prevent apoptosis and toxicity to neurons by A-beta [15]. Garlic has been shown to improve learning and memory in rats fed with fructose [16]. Aged garlic extracts reduce impairment in cognition in rats induced with A*β* [17]. Different preparations of garlic extract improve short term memory in diabetic rats [18]. According to reviewed literature, the effect of ethanol garlic (*A. sativum*) extract on the activities of hippocampus Ca^2+^ ATPase, Na^+^/K^+^ ATPase, glutamine synthetase and it link with memory in diabetic rats has not been studied. However, we have reported that hippocampus Na^+^/K^+^ ATPase and Ca^2+^ ATPase are involved in spatial working memory in normal Wistar rats [19]. We further explored the possibility of memory improvement in STZ-nicotinamide induced diabetic Wistar rats by studying the effect of ethanol garlic (*A. sativum*) extract on the activities of hippocampus Na^+^/K^+^ ATPase, glutamine synthetase, and Ca^2+^ ATPase.

## 2. Materials and Methods

### 2.1. Ethanol Garlic Extraction

The cold maceration method as described in [20] was used. Extraction was carried out at the biomedical research laboratory in Western Campus, KIU (Kampala International University), Uganda. Pieces of small garlic which weighed 500 g were peeled and later homogenized in cold 0.9% NaCl sterile solution in a volume of 70 ml. The resultant paste was then suspended for 48 hours in 80% ethanol in an air tight jar and shaken after every five minutes, 3 times a day. After 48 hours, suspension was filtrated using filter paper of Whitman type. After three times of repeated filtration, a clear filtrate was obtained. A rotary evaporator was then used to concentrate the filtrate at 40°C in a water bath. The concentrated extract was placed in a conical flask and evaporated further at 50°C in an oven, after which the extract of the study was obtained.

### 2.2. Experimental Rats

Twenty-four (24) male Wistar rats weighing 200–250 g were used. These were purchased from animal facility at MUST (Mbarara University of Science and Technology). The rats were housed in clean cages in the animal house facility at KIU Western campus which were used to house the experimental rats and then they were acclimatized to new surroundings for two weeks. Standard rat pellets were purchased from Nuvita Feeds (U) Ltd and water was provided throughout the experimental period with exception when fasting blood glucose levels were taken [21]. The experimental rats were divided randomly into three groups each having 8 rats (Table 1).

### 2.3. Induction of Hyperglycemia in Wistar Rats

Wistar rats were allowed to feed freely for five days. Sixty (60) mg/kg streptozotocin purchased from Sigma Aldrich was dissolved in 0.05 ml of citrate buffer (pH 4.5) and 120 mg/kg nicotinamide purchased from Zac's pharmacy in Bushenyi was dissolved in 0.5 ml of normal saline. Hyperglycemia was induced using a single intraperitoneal injection of 60 mg/kg STZ and after 15 minutes 120 mg/kg nicotinamide was injected intraperitoneally [22]. Elevated levels of blood glucose were confirmed 3 days after induction by collecting blood samples from the rat tail vein through a small cut. Blood glucose levels were measured using one call glucometer [23]. Rats confirmed being hyperglycemic with blood glucose concentration (≥250 mg/dl) used for the study [22].

### 2.4. Hippocampus Dependent Memory Assessment

The method described [24] with minor modifications was used to assess memory. The experimental apparatus consisted of a box made out of plywood and measured 40 cm × 640 cm × 630 cm. Saw dust was spread two inches on the floor and lighting using overhead bulb was used. The objects and arena were cleaned using 70% ethanol on each day of the experiment and reducing olfactory cues fresh bedding was provided for each day. Object location memory test was carried out for 3 days. This test was performed, before induction of hyperglycemia and then 21 days after confirmation of hyperglycemia. The experiment was carried out for three days. On day one (habituation), the rats explored the box and behavioural room for 5-minute exposures two times and no objects were placed in the arena during this day. The cage was cleaned to remove any feces and the rat was then returned after exploration. Habituation proceeded to another rat and repeated following the same arrangement after all rats had been habituated. On training day (second day), two objects which were identical measuring 1.2 × 2500 inches were placed in the box 2.5 centimetres from its wall. The objects were located in corners A and B, 2.5 cm apart. Rats explored the two objects freely for a 10-minute trial. Those rats which failed to explore the objects on day one for less than ten seconds were not included for analysis. Memory testing occurred on day 3. To test memory, object exploration time in novel versus familiar locations was done. For each trial, one of the objects was placed in the centre of the arena instead of its original location. The experiment was run similarly during training, with 10-minute trials. All trials were videotaped with VDO camcorder version-052, USA, and analyzed later by trained technician. The exploration time in seconds of objects in novel and familiar locations was recorded for each rat and later analyzed. Exploration time was scored when the rat's head or nose touched the objects. Standing, sitting, and sniffing on the objects were also scored. Novelty index in percentage was calculated as follows.

Time spent exploring object in novel location is divided by the total time spent exploring both objects in novel and familiar locations and then multiplied by 100%.

### 2.5. Collection of Hippocampus Samples and Processing

After the memory test, the rats were placed in a container with a lid containing a towel dipped in 99% diethyl ether for 2 minutes. The rats were then sacrificed, the brain was removed, and the hippocampi were excised carefully and then homogenized to obtain a homogenate which was then centrifuged to obtain a supernatant used in analysis for GS, Na^+^/K^+^ ATPase, and Ca^2+^ ATPase activities.

### 2.6. Determining Na^+^/K^+^ ATPase Activity

The method described [25] was used to analyze thirty (30) hippocampal homogenates for Na^+^/K^+^ ATPase activity. Assay mixture (mM) consisted of 50 NaCl, 30 Tris-HCl buffer (pH 7.4), 6 MgCl_2_, 0.1 EDTA, 5 KCl, and a protein concentration of 50 *μ*g. Ouabain (1 mM) was added making a concentration of 350 *μ*L. Adenosine triphosphate (ATP) was added to start the reaction to form a 3 mM concentration. The reaction was stopped 30 minutes when 50% trichloroacetic acid in a volume of 70 *μ*L was added at a temperature of 37°C. The amount Pi liberated was calorimetrically quantified according to method described [26], using a standard reference of 300 KH_2_PO_4_. The activity of Na^+^/K^+^ ATPase in Pi/min/mg of protein in nmol was determined in the absence of ouabain from the overall activity.

### 2.7. Determining Ca^2+^ ATPase Activity

The activity of Ca^2+^ ATPase in 30 hippocampal homogenates was determined according to the method described [27]. The method [26] was used to estimate Pi (inorganic phosphates). 3 MgCl_2_, 30 Tris-HCl buffer (pH 7.4), 0.1 EGTA all in mM, and 100 *μ*g of protein in the presence or absence of 0.4 CaCl_2_ in a 200 *μ*L volume formed the medium of the assay. Addition of ATP started the reaction to give 3 mM as the final volume. Fifty percent addition of trichloroacetic acid in a volume of 70 *μ*L after 60 minutes and at thirty-seven degrees Celsius stopped the reaction. The reaction was linear with concentration of protein and time. The assay consisted of control solutions, which were used in nonenzymatic adenosine triphosphate hydrolysis. The liberated Pi was calculated calorimetrically with a reference solution of KH_2_PO_4_. Ca^2+^ ATPase activity was calculated in Pi/min/mg protein in nmol by subtracting the measured activity from calcium ions absence.

### 2.8. Determining Glutamine Synthetase Activity

The method [28] was used in the enzymatic assay of glutamine synthetase. In this method, 0.1 mL homogenates solubilised in 140 mM KCl were added to 0.1 mL of the reaction mixture in mM and incubated for 15 min (37°C). The reaction was stopped by 0.4 mL addition of a solution containing (in mM) 370 ferric chloride, 200 TCA, and 670 HCl. The absorbance of the supernatant was measured at 720 nm after centrifugation and standard quantities of ferric chloride reagent treated with c-glutamyl hydroxamate were compared to the absorbance generated. Results were expressed as percentages of the control condition in mMol of gamma glutamyl hydroxamate/hr/mg protein.

### 2.9. Statistical Analysis

Results were expressed as mean ± SEM and analyzed statistically using ANOVA followed by Tukey's multiple comparison post hoc test with *P* < 0.05 considered significant.

## 3. Results

### 3.1. The Effect of Ethanol Garlic (*A. sativum*) Extract on Blood Glucose Levels

The blood glucose levels for each week in diabetic rats were higher as compared to normal control rats. There was a significant decrease in blood glucose levels of diabetic rats treated with garlic (*A. sativum*) extract as compared with diabetic control rats (Table 2). Fasting blood glucose levels are expressed as mg/dl.

### 3.2. Effect of Ethanol Garlic Extract on Novelty Index in Different Groups

The novelty index was calculated as a percentage. The novelty index in the diabetic rats which received normal saline (49 ± 0.22) was significantly reduced (*P* < 0.05) compared with that of normal control rats (55 ± 0.11) (Table 3). However, when the diabetic rats were treated with garlic (*A. sativum*), there was significant increase (*P* > 0.05) in the novelty index (61.4 ± 0.20) compared to the novelty index (49 ± 0.22) of the diabetic rats (Table 3).

### 3.3. Effect of Ethanol Garlic (*A. sativum*) Extract on Activity of Hippocampus Na^+^/K^+^ ATPase

**Figure 1:**
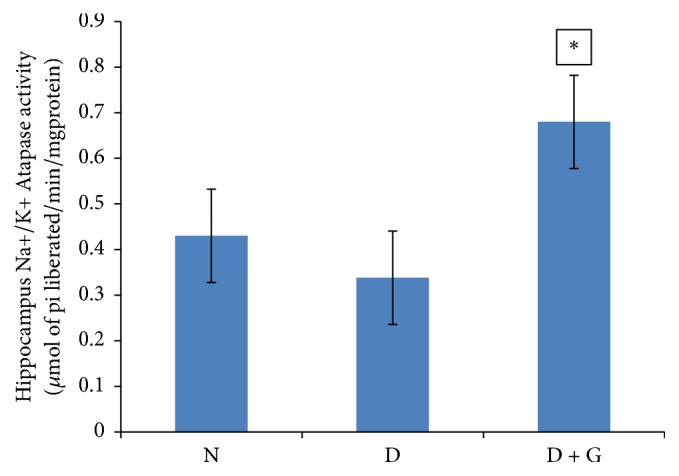
Showing effect of ethanol garlic (*A. sativum*) extract on activity of hippocampus Na^+^/K^+^ ATPase in the different groups. Each bar represents a mean of eight samples. ^*∗*^*P* < 0.05 versus diabetic control group. N = normal + saline; D = diabetic rats + saline; D + G = diabetic rats that received 1000 mg/kg of garlic (*A. sativum*) extract.

Data of hippocampus sodium-potassium ATPase activity was measured as *μ*mol of Pi liberated/min/mg protein. In diabetic controls, Na^+^/K^+^ ATPase activity (0.338 ± 0.02) was significantly decreased (*P* > 0.05) when compared with normal control rats that received normal saline (0.43 ± 0.01) (Figure 1). Administration of garlic (*A. sativum*) to diabetic rats at a dose of 1000 mg/kg resulted in a significant increase (*P* < 0.05) in hippocampus Na^+^/K^+^ ATPase activity (0.68 ± 0.01) compared to diabetic control rats (0.338 ± 0.02) (Figure 1).

### 3.4. Effect of Ethanol Garlic (*A. sativum*) Extract on Hippocampus Calcium ATPase Activity

Activity of hippocampus Ca^2+^ ATPase was calculated as *μ*mol of Pi liberated/min/mg protein.

**Figure 2:**
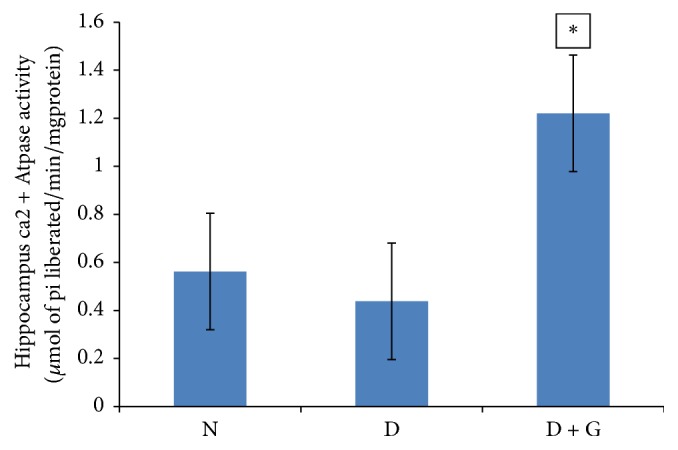
Showing the effect of ethanol garlic (*A. sativum*) extract on hippocampus calcium ATPase activity in different groups. Each bar represents a mean of eight samples. ^*∗*^*P* < 0.05 versus diabetic control group. N = normal + saline; D = diabetic rats + saline; D + G = diabetic rats that received 1000 mg/kg of garlic (*A. sativum*) extract.

Hippocampus Ca^2+^ ATPase activity in diabetic control rats (0.438 ± 0.019) decreased significantly (*P* > 0.05) as compared with normal control rats (0.56 ± 0.18) (Figure 2). Following treatment of ethanol garlic (*A. sativum*) extract to diabetic rats at a dose of 1000 mg/kg, hippocampus Ca^2+^ ATPase activity (1.22 ± 0.037) had a significant increase (*P* < 0.05) in comparison to diabetic controls (0.438 ± 0.019) (Figure 2).

### 3.5. Effect of Ethanol Garlic (*A. sativum*) Extract on Hippocampus Glutamine Synthetase Activity

**Figure 3:**
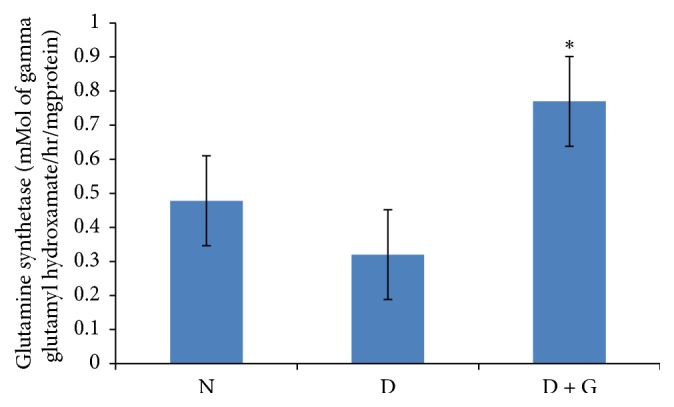
Showing effect of ethanol garlic (*A. sativum*) extract on hippocampus glutamine synthetase activity in different groups. Each bar represents a mean of eight samples. ^*∗*^*P* < 0.05 versus diabetic control group. N = normal + saline; D = diabetic rats + saline; D + G = diabetic rats that received 1000 mg/kg of garlic (*A. sativum*) extract.

Hippocampus glutamine synthetase activity was expressed as mMol of gamma glutamyl hydroxamate/hr/mg protein. Results showed that diabetic control rats recorded a significantly decreased (*P* > 0.05) hippocampus glutamine synthetase activity (0.32 ± 0.001) when compared to normal control rats (0.478 ± 0.01) (Figure 3). Treatment of diabetic rats with ethanol garlic (*A. sativum*) extract increased significantly (*P* < 0.05) hippocampus glutamine synthetase activity (0.77 ± 0.003) compared to diabetic control rats (0.32 ± 0.001) (Figure 3).

## 4. Discussion

The purpose of the current study was to determine the effect of ethanol garlic (*A. sativum*) extract on activities of hippocampus Na^+^/K^+^ ATPase, glutamine synthetase, and Ca^2+^ ATPase in streptozotocin-nicotinamide induced in diabetic Wistar rats as possible mechanisms for memory improvement. Results showed that the preference for novelty was significantly reduced in the diabetic rats, indicating impairment in memory. This result is consistent with studies [18, 29–31]. However, when the diabetic Wistar rats were treated with garlic (*A. sativum*) extract, there was an increased preference for novelty suggesting an improvement in memory. This result is in agreement with previous studies [18, 32]. Na^+^/K^+^ ATPase is important in regulating cell volume, active transport of K^+^ into the cell with outflow of Na^+^, transmembrane fluxes of Ca^2+^, and neurotransmitters release [33]. Results showed a significantly decreased activity of hippocampus Na^+^/K^+^ ATPase in rats with diabetes. This is consistent with previously reported studies where Na^+^/K^+^ ATPase activity decreased in brain of diabetic rats [31, 34]. The decreased activity of Na^+^/K^+^ ATPase could be related to increased oxidative stress in the hippocampus [35]. This leads to death of neurons due to a reduction in antioxidant defence mechanisms [36]. Administration of garlic has been shown to reduce production of reactive oxygen species [37]. In this study, treatment of diabetic rats with garlic extract increased significantly the activity of hippocampus Na^+^/K^+^ ATPase suggesting the ability of garlic to enhance functioning of this enzyme. Ca^2+^ ATPases are crucial for maintenance of intracellular calcium levels. Results showed that Ca^2+^ ATPase activity was significantly decreased in the hippocampus of diabetic rats. This is in line with studies [31, 34]. This could be due to increase in formation of lipid peroxides [38] or overload of intracellular calcium levels [39]. It has been suggested that increase in intracellular Ca^2+^ concentration occurs due to Ca^2+^ ATPase inhibition and leads to alteration in signalling pathways [40]. It has been reported that the activity of this enzyme is related to the surrounding structural and lipid environment of the synaptosomal membrane [41]. Indeed, the decrease in activity of Ca^2+^ ATPase may be due to alterations in membrane phospholipids, which is related closely to the microenvironment around the enzyme [42]. It has been suggested that S-allyl cysteine, Alliin, compounds in garlic prevent degradation of membrane lipids [43]. The significant increase in activity of hippocampus calcium ATPase after treatment of diabetic rats with garlic extract may suggest the effect of active compounds in garlic in restoration of Ca^2+^ ATPase activity. Neurotransmitter glutamate is released from glutamatergic neuronal vesicles through a calcium-dependent mechanism. Glutamine synthetase (GS) is an enzyme important in controlling the intracellular concentration of glutamate by converting it into glutamine [44]. Glutamine synthetase helps to maintain the concentration of ammonia within normal limits because excessive amounts of ammonia ions are toxic to the brain [45]. The decreased activity could be due to downregulation of the enzyme, increased clearance of glutamine synthetase, and modulation of its activity by nitric oxide [46]. GS is suggested to be vulnerable to increased protein oxidation and nitration which inhibits its activity [5, 47]. Treatment with garlic extract increased significantly the activity of hippocampus glutamine synthetase suggesting activation of this enzyme.

In conclusion, based on the results of the present study showing that ethanol garlic (*A. sativum*) extract improves memory in STZ-nicotinamide induced diabetic Wistar rats, the mechanism for this improvement could be due to increase in activities of hippocampus sodium-potassium ATPase, Ca^2+^ ATPase, and glutamine synthetase.

## Figures and Tables

**Table 1:** 

Groups	Treatments
(A) Normal control rats	Normal rats orally given 1 ml of normal saline daily.

(B) Diabetic rats	Hyperglycemic rats fed with standard pellet diet and orally given 1 ml of normal saline.

(C) Diabetic rats + 1000 mg/kg garlic	1000 mg/kg of ethanol extract of garlic administered to hyperglycemic rats daily for three weeks.

**Table 2:** 

Fasting blood glucose levels (mg/dl)				
Groups	Day 3	Day 7	Day 14	Day 21
(A) Normal control rats	110 ± 0.64	108 ± 0.58	109 ± 0.39	108 ± 0.38
(B) Diabetic rats	323 ± 19.34	344 ± 12.42	449 ± 7.41	468 ± 6.07
(C) Diabetic rats + 1000 mg/kg of ethanol garlic extract	330 ± 14.63	297 ± 21.36	143 ± 0.99	137 ± 0.41^*∗*^

Results are expressed as mean ± SEM (*n* = 8). The data was analyzed using ANOVA followed by Tukey's post hoc test. ^*∗*^*P* < 0.001 versus diabetic rats.

**Table 3:** Showing the effect of ethanol garlic extract on the novelty index in different groups.

Groups	Novelty index (percentage)
(A) Normal control rats	55 ± 0.11
(B) Diabetic rats	49 ± 0.22
(C) Diabetic rats + garlic at 1000 mg/kg b.w.	61.4 ± 0.20^*∗*^

Results are expressed as mean ± SEM (*n* = 8). ^*∗*^*P* < 0.05 versus diabetic rats.
